# Disorders of puberty and neurodevelopment: A shared etiology?

**DOI:** 10.1111/nyas.15246

**Published:** 2024-10-21

**Authors:** Jordan E. Read, Alexandru Vasile‐Tudorache, Angel Newsome, María José Lorente, Carmen Agustín‐Pavón, Sasha R. Howard

**Affiliations:** ^1^ Centre for Endocrinology, William Harvey Research Institute Queen Mary University of London London UK; ^2^ Department of Cell Biology, Functional Biology and Physical Anthropology Faculty of Biological Sciences, University of Valencia Valencia Spain; ^3^ Department of Paediatric Endocrinology Barts Health NHS Trust London UK

**Keywords:** autistic spectrum disorder, disordered puberty, hormone therapy, neurodevelopment, precocious puberty

## Abstract

The neuroendocrine control of puberty and reproduction is fascinatingly complex, with up‐ and down‐regulation of key reproductive hormones during fetal, infantile, and later childhood periods that determine the correct function of the hypothalamic–pituitary–gonadal axis and the timing of puberty. Neuronal development is a vital element of these processes, and multiple conditions of disordered puberty and reproduction have their etiology in abnormal neuronal migration or function. Although there are numerous documented cases across multiple conditions wherein patients have both neurodevelopmental disorders and pubertal abnormalities, this has mostly been described ad hoc and the associations are not clearly documented. In this review, we aim to describe the overlap between these two groups of conditions and to increase awareness to ensure that puberty and reproductive function are carefully monitored in patients with neurodevelopmental conditions, and vice versa. Moreover, this commonality can be explored for clues about the disease mechanisms in these patient groups and provide new avenues for therapeutic interventions for affected individuals.

## Graphical abstract

Disorders of puberty result primarily from dysregulation of gonadotropin‐releasing hormone secretion in the hypothalamus. Studies have uncovered associations between pubertal timing and neurodevelopment. This review discusses the overlap between the brain circuitry controlling puberty and behavior, highlights neurodevelopmental conditions with associated disordered puberty, and explores the possibility of dual treatment with hormone therapies. A better understanding of the molecular pathways controlling both conditions will enable improved diagnosis and therapeutic interventions. Figure created in BioRender. Read, J. (2024) BioRender.com/h58g149.

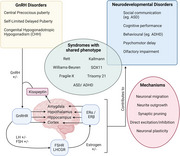

## INTRODUCTION

Puberty is the transition between juvenile and adult states, whereby an organism gains reproductive capacity and sexual maturity.^[^ ^1^ ^]^ It is a period of momentous anatomical, reproductive, and cognitive development, in parallel with the maturation of multiple neural pathways within the brain.^[^ ^2^, ^3^ ^]^ It is a time during which the structure and function of the brain regions controlling both reproductive systems and cognition change dramatically. Moreover, healthy puberty requires appropriate function of the underlying hypothalamic–pituitary–gonadal (HPG) axis, which is dependent on the development of hypothalamic neuroendocrine networks during fetal life. A complex set of positive and negative feedback interactions among these central networks, the pituitary gland, and the gonads during fetal and postnatal stages allow for the development of this axis and then, after a period of near‐dormancy in mid‐childhood, reactivation in puberty.

It is unsurprising, therefore, that research has increasingly uncovered close associations between the development of the hypothalamic gonadotropin‐releasing hormone (GnRH) neuronal networks responsible for pubertal onset and those responsible for the social, emotional, and psychomotor development of adolescence.^[^ ^4^ ^]^ Moreover, more pervasive neurodevelopmental conditions are also seen in combination with disorders of the reproductive axis in infancy, during puberty, and on into adult life. This review will explore the degree to which there is a shared etiology of pubertal and neurobehavioral conditions and the overlap in their manifestation.

## REGULATION OF THE HPG AXIS

A delicate equilibrium exists in the control of excitatory and inhibitory factors acting on the neuronal pathways of the HPG axis during childhood. The HPG axis is active during infancy and then quiescent until puberty. At puberty onset, this balance is tipped toward stimulatory inputs, triggering increased GnRH pulsatility, which results in pubertal progression.^[^ ^5^ ^]^ A key excitatory molecule, kisspeptin, is expressed in neurons of the arcuate nucleus of the mediobasal hypothalamus, and these KNDy neurons coexpress the stimulatory peptide neurokinin B and the inhibitory peptide dynorphin A. Kisspeptin, neurokinin B, glutamate, leptin, and androgens are among the known drivers to stimulate GnRH release, and dynorphin A and makorin ring finger protein 3 (MKRN3) inhibit its release, acting as the puberty brake during mid‐childhood.^[^ ^6^ ^]^ Gamma amino‐butyric acid has a more complex relationship with GnRH as, despite acting as a central nervous system inhibitor in many contexts, it has an apparent excitatory effect on GnRH neurons at the cell bodies as well as the median eminence.^[^ ^7^ ^]^ Most recently, the neuromodulator nitrous oxide (NO) and its regulator NO synthase (NOS1) have been implicated in GnRH regulation, playing an inhibitory role to regulate timely pulsatile release.^[^ ^8^ ^]^

The role of kisspeptin, encoded by *KISS1* (*Kiss1* in non‐human species) and its receptor (*KISS1R*), in the regulation of the HPG axis has been studied extensively in multiple species.^[^ ^9^, ^10^, ^11^, ^12^, ^13^ ^]^ The KNDy neurons of the hypothalamus are widely accepted to be the pulse generator controlling GnRH neuron activity.^[^ ^13^, ^14^ ^]^ During pubertal transition, a series of changes occur that lead to close anatomical–functional contact between Kiss1 and GnRH neurons. At the onset of puberty, expression of *KISS1* and *KISS1R* in the hypothalamus has been shown to increase in mice, rats, monkeys, and humans. These increased expression levels are due to changes in chromatin condensation in gene expression enhancement sites.^[^ ^15^ ^]^ This increase in kisspeptin expression is linked to increases in the amplitude and frequency of GnRH pulsatile release, thus driving HPG axis maturation.^[^ ^16^ ^]^ There is also an increase both in the number of Kiss1 neurons in the hypothalamus and their projections to GnRH neurons.

In addition, GnRH neurons become increasingly sensitive to kisspeptin and experience an increased activation of intracellular signaling cascades that are derived from KISS1R activation.^[^ ^15^, ^17^ ^]^ Together, all these changes promote a maturation of the circuits and an increase in KISS1/KISS1R/GnRH signaling during puberty.

Although the regulation of GnRH neuronal activity is central to the timely onset of puberty, GnRH neuronal migration is also a critical factor in the establishment of the HPG axis and pubertal timing. GnRH neurons originate in the nasal placode, migrating through the forebrain to the hypothalamus.^[^ ^18^ ^]^ Failure of migration has been linked to hypogonadotropic hypogonadism in many studies to date,^[^ ^19^ ^]^ whereas an excess of GnRH neurons in the hypothalamus has been demonstrated to drive premature pubertal development in mice.^[^ ^20^ ^]^ Appropriate regulation of both GnRH neuronal migration and activity is thus crucial for the physiological function of the neuroendocrine axis.

## PUBERTY AND ITS DISORDERS

Puberty involves a series of structural and physiological changes, including the acquisition of the gametogenic and endocrine potential of the gonads, an increase in the size and function of the reproductive organs, and the appearance of secondary sexual characteristics.^[^ ^21^ ^]^ Beyond reproductive maturation, individuals also experience important somatic, behavioral, and psychological changes,^[^ ^21^, ^22^, ^23^ ^]^ mainly due to the actions of sex steroids released by the ovaries and testes and also from the effects of non‐HPG hormones, such as adrenal androgens.^[^ ^24^ ^]^

Although the timing of puberty onset manifests as a spectrum in the healthy population, pubertal onset with more than two standard deviations beyond the normal range is considered disordered. Although there is some variation among different geographical locations, the accepted thresholds for the definition of precocious puberty in most of the developed world are initiation of puberty before the age of 8 years in girls and 9 years in boys. Precocious puberty can be further subdivided into central precocious puberty (CPP), due to premature maturation of the HPG axis,^[^ ^25^ ^]^ and peripheral precocious puberty (PPP), independent of GnRH activity and due to excess sex hormone production from the gonads, adrenal glands, ectopic sites, or exogenous sources.^[^ ^26^ ^]^ Delayed puberty occurs when an adolescent fails to enter puberty by the age of 13 years in girls and 14 years in boys.^[^ ^27^, ^28^ ^]^ Given the spectrum of pubertal timing, the inclusion of data from early puberty studies is also relevant to discussions within this review. Deviation from the normal age range of onset can have wide‐ranging health implications for patients. In a UK Biobank study, disordered puberty was strongly correlated to adverse later life health outcomes, including increased risk of Type 2 diabetes, cardiovascular disease, and obesity. Among adverse health outcomes, neurological, cognitive, and psychological effects were associated with both early and late pubertal onset in both sexes.^[^ ^27^ ^]^

### Central precocious puberty

CPP follows the normal physiological processes of pubertal onset but occurs at a younger than biologically desirable age. The majority of patients with CPP have isolated forms of the disease, either sporadic or familial, with no anatomical abnormalities (such as hypothalamic tumors or lesions) detected. CPP may also occur in the context of wider syndromic conditions. Both genetic and epigenetic mechanisms have been found to underlie isolated and syndromic forms of CPP.^[^ ^29^ ^]^ To date, only a small number of genetic causes of CPP have been identified. These include *KISS1/KISS1R*,^[^ ^30^, ^31^ ^]^ *MKRN3*,^[^ ^32^ ^]^ delta‐like non‐canonical Notch ligand 1 (*DLK1*),^[^ ^33^ ^]^ and Methyl‐CpG‐binding protein 2 (*MECP2*).^[^ ^34^ ^]^ Teles et al. identified an autosomal dominant variant in *KISS1R* ^[^ ^31^ ^]^ that confers prolonged expression of the receptor and thus increased kisspeptin signaling.^[^ ^35^ ^]^ Perhaps surprisingly, only a small number of variants in the genes for kisspeptin and its receptor have been described to cause disordered puberty. In these described cases, presentation of CPP was at a very young age, pointing to the importance of kisspeptin pathways for postnatal HPG axis regulation and pubertal onset.

*MKRN3* gene variants are the most commonly identified genetic cause of CPP.^[^ ^36^ ^]^ MKRN3 is a protein encoded by a maternally imprinted gene found on chromosome 15q11–q13. Due to maternal imprinting, the maternal allele is silent; thus, the paternal allele alone is expressed.^[^ ^36^ ^]^ *Mkrn3* mRNA expression in the arcuate nucleus of mice is found to fall immediately before puberty, suggesting a suppressive role of the protein in GnRH regulation.^[^ ^32^ ^]^ This is a finding confirmed in healthy human subjects due to a fall in circulating levels of MKRN3 in both sexes prior to pubertal onset.^[^ ^37^, ^38^ ^]^ MKRN3 regulates *Gnrh1* indirectly through repression of Kiss1 and Tac3.^[^ ^39^ ^]^ It is proposed that the E3 ubiquitin ligase activity of MKRN3 is integral to its role in regulating *KISS1* and *TAC3*, with variants in the zinc finger domain of the protein leading to a loss of repressor activity. Most recently, studies have demonstrated that loss of *Mkrn3* expression leads to an increase in dendritic spines in the arcuate nucleus, which has a role in regulating neuronal development and plasticity.^[^ ^40^ ^]^ Multiple nonsense, missense, and copy number variants in *MKRN3* have been identified in children with CPP, with a median age of onset of puberty in girls six years of age.^[^ ^41^, ^42^ ^]^ It is reported that *MKRN3* variants associated with CPP are not linked to cognitive impairment.^[^ ^42^ ^]^

*DLK1* is also a paternally inherited imprinted gene, encoding a transmembrane protein involved in inhibiting the Delta–Notch pathway and cellular differentiation.^[^ ^43^, ^44^ ^]^ It was first implicated in neuroendocrine regulation when it was found to be highly expressed in murine hypothalamic nuclei relevant to GnRH pathways.^[^ ^43^ ^]^ The increase in hypothalamic expression after birth is suggestive of a role in neuronal development. A number of *DLK1* variants have been identified in children with non‐syndromic CPP. In 2017, multiple members of a family with CPP were identified to have a large 14 kb deletion in *DLK1* with an additional duplication on 269 bp. These subjects had no detectable circulating DLK1, and CPP was isolated in the absence of any syndrome.^[^ ^33^ ^]^

MECP2, known primarily for its association with the neurological and developmental disorder Rett syndrome, has been explored in the context of CPP by Canton et al.^[^ ^34^ ^]^ This study identified rare heterozygous variants in *MECP2* among a cohort of 404 girls with idiopathic CPP, which included both missense mutations and insertions. None of the girls exhibited symptoms of Rett syndrome, but neurobehavioral features such as autism and microcephaly were seen. *MECP2* is expressed in hypothalamic regions associated with GnRH regulation in mice, suggesting a potential mechanism influencing pubertal onset.

In recent years, the role of epigenetic regulation of genes involved in pubertal onset has been highlighted. A study by Bessa et al. explored DNA methylation patterns in peripheral blood leukocytes in females with and without CPP. They identified more than 120 chromosomal locations at which methylation patterns differed prior to and after puberty.^[^ ^45^ ^]^ In the majority of the postpubertal females, these areas were hypermethylated and found to be on the X chromosome. Those with CPP were found to have more hypermethylated CpG sites than those of both the pubertal and prepubertal non‐CPP participants. As increased DNA methylation of promoter regions is most often associated with gene silencing, this study demonstrates how pubertal onset, both normal and precocious, involves widespread patterns of gene silencing—indicative of significant epigenetic involvement in its regulation. As methylation patterns can be influenced by environmental factors, such as nutrition and stress, these findings are suggestive of how the timing of puberty could be affected by early life and ongoing external influences and closely linking genetic predispositions with environmental regulation.

### Delayed puberty and hypogonadism

Delayed (or absent) puberty due to central hypogonadism, with deficiency of hypothalamic GnRH production and/or pituitary gonadotropins, is most commonly genetic in etiology. Over 60 genes affecting the HPG axis have been identified to contribute to the pathogenesis of central hypogonadism.^[^ ^46^ ^]^ These include factors regulating GnRH development, migration, and maturation. The most frequently identified candidates include *ANOS1*, *PROK2/PROKR2*, and *FGFR1* ^[^ ^47^ ^]^; regulators of GnRH neuronal activity (*TAC3*/*TACR3*, *KISS1*/KISS1R, NOS1)^[^ ^48^ ^]^; and genes involved in GnRH downstream function (*GNRHR*, *FSHB*, *LHB*) (Table 1). Individuals are frequently found to have associated neurodevelopmental conditions, including sensorineural hearing loss, anosmia, synkinesis (mirror movements), or hypoplasia of the corpus callosum. These conditions potentially indicate a more pervasive neuronal maturation disorder. This is particularly true in those patients with loss‐of‐function mutations in GnRH neuronal development genes.

**TABLE 1:** Details of genes related to gonadotrophin‐releasing hormone (GnRH) neuronal development and their encoded proteins and functions.

Gene symbol (human/rodent)	Encoded protein	General function
*MKRN3*/*Mkrn3*	Makorin ring finger 3	Inhibits kisspeptin release/acts as repressor of puberty initiation
*GABA*/*Gaba*	Gamma amino‐butyric acid	Neuronal inhibitor (general action)/excitatory action of GnRH neurons (specific action)
*nNOS*/*nNos*	Nitrous oxide synthase 1	Functions include synaptic plasticity in the central nervous system, central regulation of blood pressure, smooth muscle relaxation, and vasodilatation. Suggested roles in reproduction and neurodegeneration
*KISS1*/*Kiss1*	Kisspeptin	Stimulatory action on pulsatile GnRH release
*KISS1R*/*Kiss1r*	Kisspeptin receptor	G‐protein‐coupled receptor, involved in the control of GnRH release
*GnRH*/*Gnrh1*	Gonadotrophin‐releasing hormone	Stimulates release of LH and FSH, required for hypothalamic‐pituitary stimulation of gonadal function for puberty and reproduction
*GnRHR*/*Gnrhr*	Gonadotrophin‐releasing hormone receptor	G‐protein‐coupled receptor, required for GnRH action
*DLK1*/*Dlk1*	Delta‐like non‐canonical Notch ligand 1	Involved in cell growth and differentiation
*MECP2*/*Mecp2*	Methyl‐CpG‐binding protein 1	Transcriptional regulator and regulator of chromatin compaction, variants cause Rett syndrome
*TAC3*/*Tac2*	Neurokinin 3 (2 in mice)	Stimulatory action on pulsatile GnRH release
*TAC3R*/*Tac3r*	Neurokinin 3 receptor	G‐protein‐coupled receptor, involved in control of GnRH release
*ANOS1*/*Anos1*	Anosmin 1	Role in GnRH neuronal migration
*PROK2*/*Prok2*	Prokinectin 2	Role in GnRH neuronal migration
*PROKR2*/*Prokr2*	Prokinectin receptor 2	Role in GnRH neuronal migration
*FGFR1*/*Fgfr1*	Fibroblast growth factor receptor 1	Role in GnRH neuronal development
*FSHB*/*Fshb*	Follicle‐stimulating hormone beta subunit	Beta subunit of the gonadotropin hormone FSH, downstream of GnRH
*FSHR*/*Fshr*	Follicle‐stimulating hormone receptor	G‐protein coupled receptor, required for FSH action
*LHB*/*Lhb*	Luteinizing hormone beta subunit	Beta subunit of the gonadotropin hormone LH, downstream of GnRH
*LHR*/*Lhr (LH*/*CGR)*	Luteinizing hormone receptor	G‐protein coupled receptor, required for LH action
*IGSF10*/*Igsf10*	Immunoglobulin superfamily member 10	Role in GnRH neuronal migration
*HS6ST1*/*Hs6st1*	Heparan sulfate 6‐*O*‐sulfotransferase 1	Role in GnRH neuronal development and function
*CCDC141*/*Ccdc141*	Coiled‐coil domain containing 141	Role in GnRH neuronal migration
*EAP1*/*Eap1 (IRF2BPL*/*Irf2bpl)*	Interferon regulatory factor 2–binding protein like	E3 ubiquitin ligase, function as transcription activator
*BDNF*/*Bdnf*	Brain‐derived neurotrophic factor	Role in neurite outgrowth, synaptogenesis, and plasticity
*FMR1*/*Fmr1*	Fragile X mental retardation	Regulates synaptic plasticity. Variants cause fragile X syndrome
*CYFIP*/*Cyfip*	Cytoplasmic FMR1 protein	Regulation of FMR1 and synaptic remodeling, variants cause Prader–Willi syndrome
*NLGN3*/*Nlgn3*	Neuroligin 3	Role in GnRH neuronal neuritogenesis
*SOX10*/*Sox10*	SRY‐box transcription factor 10	Transcription factor involved in the regulation of embryonic development and in the determination of cell fate
*SOX11*/*Sox11*	SRY‐box transcription factor 11	Transcription regulator involved in neuronal development
*POLR3A*/*B*/*Polr3a*/*b*	RNA polymerase III subunit A/B	RNA transcription, variants associated with 4H syndrome
*PNPLA6*/*Pnpla6*	Patatin‐like phospholipase domain containing 6	Role in neurite outgrowth and neuronal differentiation
*TUBB3*/*Tubb3*	Tubulin beta 3 class III	Role in neurogenesis, axon guidance, and maintenance.

Abbreviations: FSH, follicle‐stimulating hormone; LH, luteinizing hormone.

Self‐limited delayed puberty, with temporary hypogonadism in adolescence that resolves spontaneously or with a short course of sex steroid treatment, is the most common cause of delayed puberty. Patients are, in general, otherwise healthy individuals without long‐term reproductive issues but often suffer with low self‐esteem and psychosocial distress due to their late pubertal onset.^[^ ^49^ ^]^ It is well established that disordered puberty has an impact on and is affected by mental well‐being in both sexes, with evidence of increased incidence of depression, eating disorders, and anxiety in girls and boys who experience either early or late pubertal development.^[^ ^50^, ^51^, ^52^ ^]^ These psychosomatic effects can in part be attributed to the major bodily changes experienced at puberty and a discordance in their occurrence compared to their peers. Although these may resolve with treatment leading to pubertal progression, suggesting these issues are secondary to falling behind their peers in development, these associations with disordered puberty highlight the importance of improving diagnosis, particularly in neurodivergent patients, such that appropriate support can be offered by clinicians.^[^ ^53^ ^]^

However, genetic etiology in familial self‐limited delayed puberty has also implicated both GnRH neuronal development genes (*IGSF10*, *HS6ST1*, *CCDC141*) and genes involved in the etiology of syndromic neurodevelopmental conditions. Interferon regulatory factor 2–binding protein‐like gene *(IRF2BPL)*, also known as early in puberty (*EAP1*), has been associated with delayed puberty. Mancini et al. identified two heterozygous variants in their cohort of self‐limited delayed puberty patients: an in‐frame deletion (Ala221del) and a missense variant (Asn770His).^[^ ^54^ ^]^ Both variants demonstrated a reduced ability to transactivate the GnRH promoter in vitro. Additionally, knockdown of *Eap1* results in a delay of pubertal initiation in rats and reduced expression of *GnRH*.^[^ ^55^ ^]^ Both homozygous and heterozygous variants in *IRF2BPL*, including missense, nonsense, frameshift, and truncating variants, have been associated with severe developmental and epileptic encephalopathies.^[^ ^56^, ^57^ ^]^ Thus, it is possible that a less severe mutational burden, such as with partial defects in protein function, may result in milder phenotypes of late puberty without severe hypogonadism or neurodisability. Current knowledge suggests that the genetic background of congenital hypogonadotropic hypogonadism (CHH) and self‐limited disordered puberty is a spectrum that spans from rare pathogenic variants of a small number of genes known to underlie only delayed puberty (e.g., *EAP1*), to a group that is exclusively causal in CHH (e.g., *ANOS1*), with a large area of overlap between these ends of the spectrum where variants in genes (e.g., *GNRHR*) are involved in the etiology of both delayed and absent puberty.^[^ ^58^ ^]^

Primary gonadal insufficiency leading to pubertal delay or failure is most commonly due to chromosomal aberrations, including Turner and Klinefelter syndromes.^[^ ^59^ ^]^ Here the overlap with neurobehavioral difficulties is well described and includes associations with autism, attention‐deficit, and impaired social and language skills.^[^ ^60^, ^61^ ^]^

Whether treatment of delayed puberty with sex steroids could lead to an alleviation of neurodevelopmental phenotypes is difficult to discern due to the inability to determine how treated patients would have developed without treatment. Evidence in mice suggests exogenous sex steroids can have a restorative effect. Hypogonadal female mice demonstrated a return to normal female sexual behavior upon administration of estrogen and progesterone.^[^ ^62^ ^]^ In clinical reports, testosterone administration for Klinefelter syndrome has been reported to reduce tremor.^[^ ^63^ ^]^ In one patient with hypogonadotropic hypogonadism, autism, and myoclonus, restoration of normal range testosterone resulted in the resolution of myoclonus.^[^ ^64^ ^]^ Estrogen has been shown to impact memory and cognition by modulating synaptic plasticity. In the female rat brain, estrogen has been shown to stimulate hippocampal neuron proliferation, suggesting that exogenous sex hormones may play a role in modulating non‐reproductive brain functions.^[^ ^65^ ^]^ The neuroprotective effects of estrogen make it an attractive candidate as a dual modal treatment for hypogonadotropic hypogonadism and autism in girls.^[^ ^66^ ^]^

## INFLUENCE OF PUBERTY ON NEURODEVELOPMENT

The link between the onset of puberty and the remodeling of brain circuits involved in cognition and behavior has long been reported and has been recently reviewed.^[^ ^67^, ^68^, ^69^ ^]^ Although puberty and adolescence are distinct entities, the hormonal changes of puberty have actions in the brain that contribute to the behavioral changes that occur during adolescence.^[^ ^70^ ^]^ It has been shown that at the time of puberty, cortical gray matter volume begins to decline.^[^ ^4^ ^]^ The cause of this decline is thought to be synaptic pruning—the removal of redundant synapses within the brain that occurs throughout development. Synaptic pruning during puberty is supported in mouse and rat models, though its quantification is limited by cell‐type specificity and sexual dimorphism.^[^ ^71^ ^]^ Experiments with rat models demonstrate that the onset of puberty in both males and females influences synapse density in the medial prefrontal cortex.^[^ ^72^ ^]^ Myelination of neurons in cortical regions of the brain is shown to increase during adolescence and linked to the development of cognition, learning, and behavior. In subcortical gray matter and several cortical regions, this increase in myelination has been shown to correlate closely with pubertal stage, further supporting a link between puberty and adolescence.^[^ ^73^, ^74^ ^]^

For the neuronal and hormonal changes of puberty to play a role in neurodevelopment, a crosstalk between the distinct brain regions responsible for each is required. Although GnRH and KNDy neurons reside in the hypothalamus, it is well established that telencephalic regions are highly involved in the control of behavior and social development. Some evidence suggests a role of kisspeptins in regulating social behavioral responses. In mice, two populations of Kiss1 neurons are present: a hypothalamic population responsible for regulation of reproduction and a second population in the medial nucleus of the amygdala, a region responsible for regulation of social behavior.^[^ ^75^ ^]^ Specifically, an extended role for kisspeptin regulating luteinizing hormone (LH) secretion through its action in the amygdala has been reported, suggesting that its role in regulating pubertal timing reaches beyond the control of GnRH release.^[^ ^76^ ^]^ Studies by Comninos et al. found kisspeptin to alter resting brain connectivity, resulting in the enhancement of sexual and emotional processing.^[^ ^77^ ^]^ It is therefore suggested that kisspeptin neurons may be responsible for regulating both reproductive circuits in the hypothalamus and social circuits in extra‐hypothalamic regions of the brain.

Additionally, the hypothalamic increase in Kiss1 expression in wild‐type mice is shown to be mirrored in the hippocampus.^[^ ^78^ ^]^ In mice with severe combined immunodeficiency, this increase in expression, thought to regulate hippocampal plasticity, is absent. These mice demonstrate a schizophrenia‐like phenotype in later life, with defects in sensorimotor gating. The phenotype has been shown to be partially ameliorated by the injection of Kiss1‐derived peptides, leading to the conclusion that an increase in kisspeptin expression around the time of puberty may modulate hippocampal circuits as well as those in the hypothalamus.^[^ ^78^ ^]^

Receptors for sex hormones have been mapped in multiple regions of the brain and thus likely play a role in regulation of a diverse range of neuroregulatory functions. Pyramidal cells throughout the hippocampus and dentate gyrus have been shown to express follicle‐stimulating hormone (*FSH*) and its receptor (*FSHR*).^[^ ^79^ ^]^ *Gnrhr* expression has been reported in the cerebral cortex and hippocampus of mice and rats, with proposed roles in estrogen synthesis, aromatase regulation, and neuronal plasticity.^[^ ^80^, ^81^ ^]^ The cerebellum, responsible for voluntary motor control, has been shown to express the GnRH receptor (in mice) and LH receptor mRNA (in rats).^[^ ^82^, ^83^ ^]^ Estrogen receptors (ER‐alpha and ER‐beta) have been identified to be widespread throughout the female rat brain, suggesting diverse roles beyond regulating reproductive function.^[^ ^84^ ^]^ Estradiol in the brains of women with epilepsy has been shown to promote seizure occurrence,^[^ ^85^ ^]^ and seizure control is often noted to decline at puberty. In contrast, the role of estrogens and androgens within the cerebellum is predicted to be neuroprotective,^[^ ^86^ ^]^ perhaps suggesting that the link between disordered puberty and neurological conditions is not necessarily defined by the sex steroid but at the tissue‐specific level.

Several studies have identified an overlap between the distribution of GnRH neurons in brain regions responsible for the control of sexual development and those required for behavioral and cognitive function.^[^ ^87^, ^88^ ^]^ GnRH receptors identified on neurons of the cerebral cortex of both mouse embryos and adults are suggestive for a role of GnRH beyond the control of reproduction.^[^ ^89^ ^]^ In vitro studies demonstrate that GnRH stimulates neuronal outgrowth and increases the length of neurites in these cerebral cortical neurons.^[^ ^89^ ^]^ The same findings have been found in neuroblast cell cultures receiving exogenous GnRH,^[^ ^90^ ^]^ and GnRH has been shown to excite cortical neurons.^[^ ^91^ ^]^ Furthermore, in vivo murine studies utilizing designer receptors exclusively activated by designer drug vector technology demonstrated that inhibition of activity of hippocampal GnRHR‐expressing neurons reduced olfactory and cognitive performance.^[^ ^80^ ^]^ It is possible therefore that GnRH production upon onset of puberty also modulates neuronal plasticity in the cortex.

Indeed, sex steroids exert both organizational and activational effects in the so‐called social behavior network of the brain,^[^ ^92^ ^]^ which includes both hypothalamic and telencephalic nuclei,^[^ ^93^ ^]^ and influence social and sexual responses in a sex‐dependent manner in animal models.^[^ ^94^, ^95^, ^96^ ^]^ This brain network is enriched in neuropeptides such as oxytocin and vasopressin, traditionally hypothesized to be dysregulated in autistic conditions^[^ ^97^, ^98^, ^99^, ^100^ ^]^ and proposed as targets to treat autistic features^[^ ^101^ ^]^ (but see Ref. [103]). Evidence from mouse models on neurodevelopmental disorders causing both autism‐like features and neuroendocrine dysregulation suggest a direct impact of mutations in causal genes and an indirect impact of dysregulated hormonal levels at appropriate time points on the neurodevelopment of neuropeptide social brain circuits.^[^ ^96^, ^103^ ^]^ Thus, the normal progression of puberty shapes not only reproductive capacity but also tunes the brain for controlling typical social responses, and its dysregulation could contribute to the development of atypical social and emotional responses, such as the ones associated with autism spectrum disorders (ASDs).

### Overlap between disordered puberty and neurodevelopmental traits

Disordered puberty occurs in the context of several neurodevelopmental disorders associated with autistic features, suggesting a link between the neurodevelopmental circuits that control puberty onset and social brain activity. Multiple studies report associations between neurodevelopmental disorders and CPP (Table 2). In a study of children with preexisting medical conditions and concurrent CPP, the most common association was with conditions that led to psychomotor delay and psychiatric abnormalities, including developmental disorders and ASD.^[^ ^104^ ^]^ Rates of CPP were highest in patients previously diagnosed with ASD (16%), psychomotor delay (48%), and epilepsy (20%).^[^ ^104^ ^]^ Chromosomal duplication was identified in 50% of patients with psychomotor delay within this CPP cohort, suggesting a significant genetic component to the pathogenicity.

**TABLE 2:** Conditions reported with neurodevelopmental phenotypes associated with precocious, delayed, or both precocious and delayed puberty.

Associated syndrome, gene, or chromosomal abnormality	Primary findings	Author	References

Conditions with precocious puberty associated with NDD			
Rett syndrome	CPP, microencephaly, hypotonia, learning difficulties, abnormal EEG (1 girl)	Bas et al.	^[^ ^77^ ^]^
	CPP, microencephaly, hypotonia, learning difficulties, abnormal EEG (1 girl)	Holm	^[^ ^75^ ^]^
	CPP, developmental regression, learning difficulties, and characteristic hand movements (1 girl)	Huppke et al.	^[^ ^76^ ^]^
	CPP, seizures, and abnormal EEG (1 girl)	Yang et al.	^[^ ^78^ ^]^
	CPP, developmental regression, learning difficulties, abnormal EEG, and seizures (2 girls)	Bernstein et al.	^[^ ^79^ ^]^
*MECP2* gene variants (without Rett syndrome)	CPP caused by rare exonic variants in *MECP2* (5 girls, 4 unrelated families)	Canton et al.	^[^ ^26^ ^]^
Williams–Beuren syndrome	CPP (2 boys, 1 girl), short stature (28.3% of 101 patients), hyperthyroidism, hypercalcemia	Kim et al.	^[^ ^93^ ^]^
	CPP in 18.3% of girls (of 171 girls)	Partsch et al.	^[^ ^94^ ^]^
	CPP and advanced bone age (1 girl)	Douchi et al.	^[^ ^95^ ^]^
Fragile X syndrome (*FMR1* gene variants)	CPP, advanced bone age, learning difficulties, speech disturbance, and hyperactivity (1 girl)	Butler and Najjar	^[^ ^97^ ^]^
	CPP, delayed motor development, and characteristic features (1 girl)	Moore et al.	^[^ ^98^ ^]^
*NRXN1* gene deletions	CPP and early onset developmental delay, epilepsy, gastroesophageal reflux (2 sisters)	Harrison et al.	^[^ ^125^ ^]^
Deletion of 9p	CPP and learning difficulties, dysmorphism (2 girls, 1 boy)	Funderburk Eshel et al. Cisternino et al.	^[^ ^126^ ^]^ ^[^ ^127^ ^]^ ^[^ ^128^ ^]^
1p36 deletion syndrome	CPP and learning difficulties, epilepsy, growth delay, congenital heart defects, characteristic facial appearance (1 girl)	Kurosawa et al.	^[^ ^129^ ^]^
Triple X syndrome	CPP and learning difficulties in all patients (2 girls, 1 boy)	Grosso et al.	^[^ ^130^ ^]^
Inverted duplicated chromosome 15 syndrome	CPP and learning difficulties, epilepsy, behavioral problems, malformations (2 girls of 10 patients)	Grosso et al.	^[^ ^131^ ^]^
Maternal uniparental disomy for chromosome 14	CPP and developmental delay, growth retardation, hypotonia, scoliosis (1 boy)	Temple et al.	^[^ ^132^ ^]^

Conditions with delayed/absent puberty associated with NDD			
CHH/Kallmann syndrome	Severe HH with anosmia, hearing loss, synkinesis, learning difficulties, and social communication disorder (*ANOS1* gene variants)	Dawson et al. Jiang et al. Pingault et al. Margolin et al. Jongmans et al.	^[^ ^108^ ^]^ ^[^ ^109^ ^]^ ^[^ ^110^ ^]^ ^[^ ^111^ ^]^ ^[^ ^112^ ^]^
*SOX11* syndrome	HH (21%) developmental delay, microcephaly, and short stature	Al‐Jawahiri et al.	^[^ ^113^ ^]^
*EAP1* (*IRF2BPL*) gene variants	Self‐limited delayed puberty with severe developmental and epileptic encephalopathies	Mancini et al.	^[^ ^43^ ^]^
*NLGN3* gene variants	Delayed puberty, partial HH with ASD, and/or developmental delay	Oleari et al.	^[^ ^105^ ^]^

Conditions with both precocious and delayed/absent puberty with NDD			
Trisomy 21	CPP (2 boys, 1 girl), hypothyroidism (70%), short stature, obesity. Pubertal onset earlier in both sexes compared to general population.	Erdoğan and Güven	^[^ ^121^ ^]^
	GnRH deficiency	Manfredi‐Lozano et al.	^[^ ^122^ ^]^
Prader–Willi syndrome	Delayed puberty in 5/9 boys, small penis, and testes in 7/13 boys	Nowicki et al.	^[^ ^99^ ^]^
	CPP in patients with Prader‐Willi syndrome (1 boy) and growth hormone deficiency (1 boy), treated with GnRH (1 girl), and growth hormone (1 girl)	Vanelli et al. Pusz and Rotenstein Crino et al. Lee and Hwang	^[^ ^133^ ^]^ ^[^ ^134^ ^]^ ^[^ ^135^ ^]^ ^[^ ^136^ ^]^
Autism spectrum disorder (ASD)	Increased incidence of precocious puberty in children with ASD	Geier and Geier	^[^ ^100^ ^]^
	Precocious puberty and accelerated progression in males	Corbett et al.	^[^ ^101^ ^]^
	HH and neurodevelopmental delay	Ohlsson Gotby et al.	^[^ ^104^ ^]^
	*NLGN3* variants in patients with ASD and HH (2 patients)	Oleari et al.	^[^ ^105^ ^]^

Abbreviations: CPP, central precocious puberty; EEG, electroencephalogram; HH, hypogonadotropic hypogonadism; NDD, neurodevelopmental disorder.

*Source*: Adapted from Ref. [105].

A second large cohort study of patients with CPP aimed to identify associated anomalies. In patients identified to have CPP caused by pathogenic or predicted pathogenic variants, most were overweight or obese. Small for gestational age and short stature were also common findings. Overall, 18% of patients with CPP had multiple comorbidities, namely, disordered growth, metabolic abnormalities, and neurocognitive abnormalities, suggesting overlap between genetic controls of these key developmental processes.^[^ ^29^ ^]^ Similarly, Wannes et al. identified that in their cohort of patients with CPP without an organic etiology, 24% also presented with ASD and/or encephalopathy, and 11% had other conditions, including trisomy 21 and Williams–Beuren syndrome.^[^ ^105^ ^]^ Moreover, children with neurodevelopmental conditions frequently have altered body composition, often presenting either with undernutrition or as overweight compared to their healthy peers,^[^ ^106^ ^]^ which is well documented to impact pubertal timing. Although this can be attributed to associated disordered eating, reduced social behaviors, and lack of oromotor development, the pathogenic associations between pubertal timing and multiple neurodevelopmental conditions have been increasingly defined. It has long been known that metabolism and both pubertal timing and reproductive capacity are tightly linked, and this has recently been comprehensively reviewed.^[^ ^107^ ^]^ The crosstalk among metabolism, weight regulation, neurobehavior, and the reproductive axis is likely to be multi‐directional, and etiological drivers are difficult to define.

Neurobiologically, it is hypothesized that stress‐regulating catecholamines, such as noradrenaline, and stress‐related hormones, such as cortisol, have a permissive role in puberty regulation and onset. Studies that have indicated this include Ivanisević‐Milovanović et al., which found increased concentrations of noradrenaline in the hypothalamus of rats with precocious puberty, as well as Qi et al., which identified higher levels of urine metanephrines in samples from girls with CPP compared to a control group.^[^ ^108^, ^109^ ^]^ Furthermore, a human study, by Ergür et al., found that rates of precocious puberty were greater in children treated for attention‐deficit hyperactivity disorder (ADHD) with methylphenidate—a medication that increases synaptic catecholamine concentrations.^[^ ^110^ ^]^ Effects of medications used to treat ADHD may also influence the HPG axis directly; for example, rats treated with methylphenidate demonstrated pubertal disruption, with male rats experiencing early pubertal onset and female rats experiencing delayed onset, compared to control groups. In addition, sex hormone levels were perturbed, and histological analysis of the gonads was abnormal in both sexes. The effects of methylphenidate were transient, with a recovery of normal parameters when treatment was ceased for 30 days.^[^ ^111^ ^]^ These effects may also be mediated via dopamine pathways, as central dopamine is also increased by methylphenidate administration^[^ ^112^ ^]^; however, apart from in teleosts, dopamine has not been linked directly with pubertal timing.^[^ ^113^ ^]^ Boys prescribed stimulants for ADHD from a mean age of 7.3 years (±1.9 years, range 3.5–10.9 years) demonstrate a normal age of onset of puberty, but by 14 years of age, pubertal development was shown to be slow compared to healthy individuals.^[^ ^114^ ^]^ Studies that have linked international adoption to increased rates of CPP have brought into question the “psychosocial acceleration hypothesis,” whereby individuals raised in adverse environments are theorized to reach reproductive maturity at a younger age to enhance reproductive fitness.^[^ ^115^, ^116^ ^]^ Multiple studies have observed how psychological stress can accelerate pubertal onset—including domestic abuse in the home, environmental catastrophes, and sexual or physical abuse.^[^ ^115^ ^]^ However, the mechanisms underlying the effect of such environmental influences may include a direct hypothalamic crosstalk between corticotropin‐releasing hormone and kisspeptin pathways rather than via catecholamines.^[^ ^117^ ^]^

## NEURODEVELOPMENTAL DISORDERS WITH DISORDERED PUBERTY

An overview of these disorders is presented in Table 2, with details of specific syndromes and associations given in the following sections.

### Rett syndrome

Rett syndrome is a severe neurodevelopmental disorder caused by loss‐of‐function variants in the X‐linked gene *MECP2*. Rett syndrome presents as normal infantile development followed by developmental regression, typically by 18 months of age. Characteristics include autistic traits and loss of acquired skills, including speech and limb control.^[^ ^118^ ^]^ The X‐linked inheritance of *MECP2* means that Rett syndrome is observed almost exclusively in girls and presents with a spectrum of severity. This is largely due to the cell‐specific expression of heterozygous *MECP2* variants and mosaicism due to X inactivation.^[^ ^119^ ^]^ Several case studies have reported patients with Rett syndrome presenting with precocious puberty, suggesting a dual role for MECP2 in regulating the GnRH neuroendocrine network and socio‐behavioral neuronal circuits.

In 1985, Holm reported the first case of CPP in a female with Rett syndrome with developmental regression, mental retardation, stereotyped movements, microcephaly, abnormal electroencephalogram, and episodic hyperventilation.^[^ ^120^ ^]^ Since this report, multiple female Rett syndrome patients have been identified with precocious puberty. A number of variants have been identified, including a nonsense truncating variant, two missense variants, a 10 base pair deletion, and a larger 40 base pair deletion resulting in a frameshift. All patients presented with CPP alongside the classical Rett syndrome phenotype of developmental regression, mental retardation, and characteristic hand movements.^[^ ^121^, ^122^, ^123^, ^124^ ^]^ Timing of pubertal onset was reported to range from 6 to 10 years of age.

The most recent data from Pepe et al. aimed to investigate patients with Rett syndrome for comorbidities, including endocrine disruption.^[^ ^125^ ^]^ In a cohort of 50 patients (46 females), 74.5% of whom had pathogenic variants in *MECP2*, almost half of patients experienced menstrual cycle disruption, including oligomenorrhea and secondary amenorrhea. Precocious puberty was reported in 15.4% of patients. Although precocious onset of puberty is reported in patients with Rett syndrome, Killian et al. found that delayed menarche was experienced by 19% of patients in their cohort. Timing of menarche relative to onset of puberty was associated with severity of mutation and clinical presentation.^[^ ^126^ ^]^

MECP2 has indeed been shown to regulate the expression of genes known to play key roles in the neuronal biology of both the pubertal axis and behavioral circuits. Expression of *Mecp2* in the frontal cortex of mice was shown to follow circadian patterns. Cyclic *Mecp2* expression was found to correlate to *Dlk1* expression levels, suggesting a role for Mecp2 in positively regulating *Dlk1* RNA expression in the frontal cortex^[^ ^127^ ^]^; thus, a loss of Mecp2 could lead to reduced Dlk1 expression and thus de‐repression of GnRH neurons. Conversely, Dlk1 was shown to be upregulated in Mecp2 knockout mice in the cortex, midbrain, and cerebellum.^[^ ^128^ ^]^ There is some evidence of Dlk1 expression in rat hypothalamic arcuate and paraventricular nuclei, but a lack of direct evidence of co‐expression of Dlk1 and GnRH.^[^ ^129^ ^]^ Although a role for DLK1 in Rett syndrome has not yet been identified, it plays a known role in neurogenesis through the NOTCH and bone morphogenetic protein signaling pathways, and loss of Dlk1 expression in adult mouse subgranular zone neurons leads to abnormal hippocampal neurogenesis and cognitive impairment.^[^ ^130^, ^131^ ^]^

*MECP2* expression inversely correlates to brain‐derived neurotrophic factor (*BDNF*) and somatostatin RNA expression levels in the frontal cortex of mice.^[^ ^127^ ^]^ BDNF is reported to play roles in both behavioral neuronal axes and the gonadotropic axis. GnRH neurons extend through brain regions rich in BDNF during their development, and BDNF is shown to promote neurite outgrowth, suggesting a potential neurotropic role in GnRH neurons.^[^ ^132^ ^]^ In sheep, BDNF infusion stimulates expression of GnRH, neurokinin B, and kisspeptin.^[^ ^133^ ^]^ The role for BDNF as a neurotrophic factor is recapitulated and well reported in behavioral and social neuronal networks, with serum BDNF levels in children with ASD and psychiatric disorders elevated compared to age‐matched controls.^[^ ^134^, ^135^ ^]^ It has a well‐established role in regulating neuronal development, synaptogenesis, and plasticity, with BDNF dysfunction leading to neurodevelopmental and neurodegenerative conditions, including Rett syndrome.^[^ ^136^ ^]^

### Williams–Beuren syndrome

**FIGURE 1:**
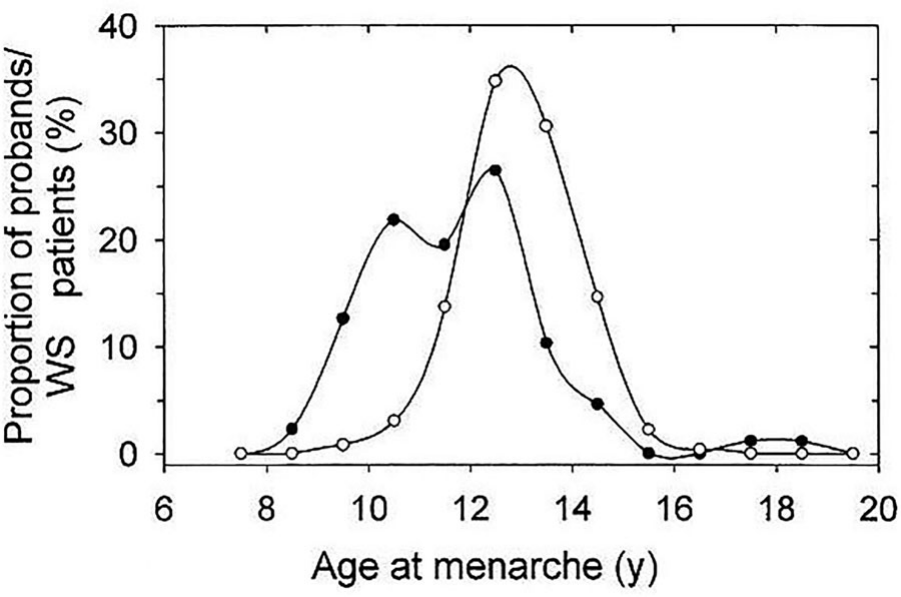
Distribution of age of menarche in girls with Williams syndrome (*n* = 86, closed circles) with healthy girls from northern Germany (*n* = 759, open circles). Distributions were significantly different (*p* < 0.001), with two peaks in the population of girls with Williams syndrome, corresponding to early puberty and normal puberty. *Source*: Partsch et al.^[^ ^94^ ^]^

Williams–Beuren syndrome is a rare disease caused by a hemizygous microdeletion of chromosome 7q.11.23. Characteristics include global cognitive impairment, dimorphic facial features, urinary tract and renal abnormalities, and supravalvular aortic stenosis.^[^ ^137^ ^]^ Approximately half of children with Williams–Beuren syndrome experience early puberty, but the incidence of true CPP is significantly lower.^[^ ^137^ ^]^ Girls and boys with Williams–Beuren syndrome in general experience earlier puberty, and boys experience an earlier growth spurt than the general population.^[^ ^138^ ^]^ A case report from 1999 reported CPP in a girl aged 9 years with Williams–Beuren syndrome. Her puberty began at age 5.5 years, and regular menstruation had started by the age of 8.5 years. Bone age was advanced to 14 years, and breast and pubic hair development was Tanner stage IV by age 9 years.^[^ ^139^ ^]^ Partsch et al. reported CPP in a German cohort of female patients with Williams–Beuren, with 18% presenting with thelarche before the age of 8 years and/or menarche before the age of 9 years^[^ ^138^ ^]^ (Figure 1). The molecular link between Williams–Beuren syndrome and the incidence of precocious puberty remains elusive.

### Fragile X syndrome

Fragile X syndrome affects 1 in 7000 males and 1 in 11,000 females worldwide.^[^ ^140^ ^]^ The syndrome is caused by a specific amplification of a CGG trinucleotide in the Fragile X mental retardation (FMR1) gene, resulting in promoter hypermethylation. It is the most common monogenic cause of intellectual disability and ASD.^[^ ^141^ ^]^ Multiple case studies of children with Fragile X syndrome presenting with precocious puberty are reported in the literature. Fragile X syndrome with precocious puberty was reported in a girl of 8.5 years old.^[^ ^142^ ^]^ Fragile X syndrome in this child presented with learning difficulties, speech disturbance, and hyperactivity. Idiopathic precocious puberty at age 6 was confirmed, with advanced bone age, breast Tanner stage 4 development, and pubertal LH and FSH responses to GnRH stimulation. In 1990, another case was reported in a 2‐year‐old girl with delayed motor development and characteristic features of Fragile X syndrome, confirmed with cytogenic studies. Breast development and skeletal development were advanced, ovaries and uterus were large for her age, and GnRH stimulation resulted in elevated serum LH and FSH concentrations, confirming CPP.^[^ ^143^ ^]^ This was the first case associating *FMR1* variants with precocious puberty.

In contrast, for patients with a Prader–Willi syndrome phenotype of Fragile X syndrome, delayed puberty is common. In a study of 13 male patients, small penis and/or testes were reported in 7 patients, with 5 of the 9 who had entered puberty reporting delayed puberty. Cytoplasmic FMR1 protein (CYFIP) expression was reduced in patients with a Prader–Willi phenotype of Fragile X syndrome, as compared to individuals presenting with either syndrome separately. ASD characteristics were reported in all patients, with a full diagnosis in over half. CYFIP1 is a protein with a known role in regulating FMR1 and synaptic remodeling.^[^ ^144^ ^]^ This could suggest a dual role in the regulation of behavioral neuronal networks and GnRH neuronal networks.

### Autism spectrum disorder

ASD is characterized by stereotypic behaviors and defects in social, communication, and behavioral traits.^[^ ^145^, ^146^ ^]^ Children with ASD demonstrate a higher than usual prevalence of precocious puberty, suggesting co‐regulation of neural circuits controlling social behavior and neuroendocrine networks.^[^ ^145^ ^]^ Studies of pubertal progression in adolescents with ASD compared to the neurotypical population demonstrated earlier initiation of puberty and faster progression, the latter more prevalent in male ASD patients.^[^ ^146^ ^]^ The genetic link between ASD and CPP has been described in case studies, including a patient with Phelan–McDermid syndrome (22q13.3 deletion), characterized by developmental delay and ASD.^[^ ^147^ ^]^ Individuals with this syndrome are also frequently reported to experience cryptorchidism (undescended testes) and accelerated growth.^[^ ^148^ ^]^

**FIGURE 2:**
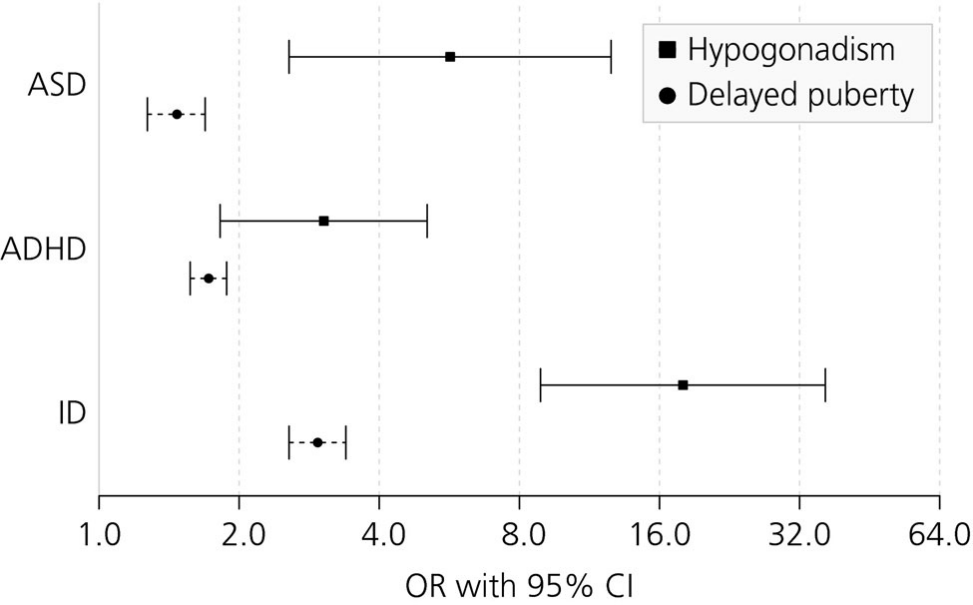
Risk of neurodevelopmental conditions in patients with hypogonadotropic hypogonadism and delayed puberty in a Swedish national cohort compared to matched controls. Shown are risks of autism spectrum disorder (ASD), attention‐deficit hyperactivity disorder (ADHD), and intellectual disability (ID) represented as odds ratios and corresponding 95% confidence intervals. CI, confidence intervals; OR, odds ratio. *Source*: Ohllson Gotby et al.^[^ ^104^ ^]^

In contrast, patients with delayed puberty due to hypogonadotropic hypogonadism (secondary to GnRH or gonadotropin deficiency) are reported to be at a higher risk of developing ASD and ADHD (Figure 2),^[^ ^149^ ^]^ demonstrating that the link between ASD and pubertal timing is not unidirectional and that neuroendocrine and behavioral neuronal circuits may be influenced by a common mechanism.

Recently, loss‐of‐function variants in *NLGN3* have been identified in patients with delayed puberty with partial hypogonadotropic hypogonadism in conjunction with ASD and/or developmental delay.^[^ ^150^ ^]^ In a cellular model, loss of NLGN3 was demonstrated to impair GnRH neuronal neuritogenesis, suggesting that this synaptic protein may be important for neuronal network formation required for both GnRH biology and cognitive and social development.

### Congenital hypogonadotropic hypogonadism/Kallmann syndrome

CHH results from low levels of GnRH production or failure of pulsatile secretion leading to delayed or absent puberty and subsequent infertility. More than 50 genes have been implicated in CHH, making diagnosis and treatment challenging.^[^ ^151^, ^152^ ^]^ Approximately 50% of patients with CHH have Kallmann syndrome, resulting from a failure of GnRH neuronal migration during embryonic development. X‐linked Kallmann syndrome due to *ANOS1* mutations is typified by severe hypogonadotropic hypogonadism and associated with a loss of the sense of smell, hearing loss, synkinesis, learning difficulties, and social communication disorder.^[^ ^153^, ^154^ ^]^

A number of heterozygous loss‐of‐function variants in *NOS1* have been identified in patients with CHH.^[^ ^48^ ^]^ These patients also present with phenotypes, including intellectual disability, anosmia, and hearing loss. These results were recapitulated in *Nos1*‐deficient mice that demonstrated defects in sexual maturation, impaired cognition, hearing, and olfaction compared to their wild‐type counterparts. Reproductive and cognitive phenotypes were rescued by NO treatment, specifically during the window of development corresponding to mini‐puberty, suggesting a temporally sensitive role for *NOS1* in regulating neural circuitry, with consequences for reproductive, cognitive, and sensory functions. These findings, and other recent studies, point to mini‐puberty as a critical developmental stage for determining both disorders of neurodevelopment and fertility in later life.^[^ ^155^ ^]^

There are also numerous syndromic conditions associated with hypogonadotropic hypogonadism, including Gordon Holmes syndrome, CHARGE syndrome, and the soxopathies (*SOX10* and *SOX11* deficiency).^[^ ^156^, ^157^, ^158^, ^159^ ^]^ Defects in many of these genes are also associated with phenotypes of neurodisability, such as in hypomyelinating leukodystrophy‐8 with hypogonadotropic hypogonadism (4H syndrome) due to mutations in *POLR3A*/*B*, or secondary to *PNPLA6* deficiency, or to *TUBB3* deficiency.^[^ ^160^, ^161^, ^162^ ^]^

### SOX11 syndrome

Pathogenic variants in *SOX11* have recently been demonstrated to underlie a neurodevelopmental disorder with developmental delay, microcephaly, and short stature, with hypogonadotropic hypogonadism found in 21% of the patient cohort.^[^ ^159^ ^]^ *SOX11* is highly expressed both in hypothalamic neural progenitor cells^[^ ^163^ ^]^ and in the majority of hypothalamic GnRH neurons in adult mice.^[^ ^164^ ^]^ *SOX11* is also highly enriched in the immortalized GnRH‐producing GT1 neuronal cell line,^[^ ^164^ ^]^ and Sox11 in a mouse model is required for both embryonic and adult neurogenesis.^[^ ^165^ ^]^ Moreover, SOX11 is known to specifically augment transcriptional activation of the *GnRH1* gene by binding to SOX‐binding sites located in the first intron (intron A) region of GnRH1. In turn, suppression of *SOX11* expression significantly decreases *GnRH1* expression, as well as GnRH secretion.^[^ ^164^ ^]^ *SOX11* is also highly expressed in the developing pituitary gland.^[^ ^159^ ^]^ A common DNA methylation signature, with an overall hypomethylation pattern, was observed for individuals with loss‐of‐function variants in *SOX11*, consistent with the role of this protein as a key epigenetic regulator for multiple neuronal circuits controlling brain development.^[^ ^159^ ^]^

### Trisomy 21

Trisomy 21 (also called Down syndrome [DS]) results from an extra copy of Chromosome 21. It is the most common cause of cognitive impairment in the population.^[^ ^166^ ^]^ Endocrine disorders are common in patients with DS, with characteristic short stature, obesity, Type 1 diabetes, hypothyroidism, and infertility.^[^ ^167^ ^]^ Incidence of disordered puberty in patients with DS is not well documented. Although studies suggest later breast development but earlier menarche in girls with DS compared to the general population, as well as later testicular development in boys, ages are not reported to be outside the normal range for pubertal onset.^[^ ^167^ ^]^ Disordered puberty in DS patients most often manifests as delayed puberty. PPP, associated with DS‐associated hypothyroidism, is reported in a few cases.

## THERAPEUTIC POTENTIAL IN NEURODEVELOPMENTAL DISORDERS

A link between the cognitive defects characteristic of DS and GnRH neuronal networks has been proposed. As described, GnRH neuronal networks extend into brain regions responsible for control of cognitive function.^[^ ^87^, ^88^ ^]^ In very recent studies, treating DS mouse models with pulsatile GnRH led to a reversal of cognitive and olfactory defects. It is proposed that both the cognitive and neuroendocrine networks within the brain are under the control of a microRNA gene network, the dysfunction of which may perturb both neurological networks.^[^ ^80^ ^]^ Together, these studies demonstrate a potential for treatment with GnRH to improve neurocognition, but further research to establish the efficacy and safety of such therapies is needed.

Interestingly, recent discoveries in patients experiencing long COVID following exposure to the SARS‐CoV‐2 virus implicate GnRH as a key player in persistent neuropathology. Infected men experience hypogonadism similar to that experienced with GnRH deficiency. Postmortem investigation of brain tissue of patients who had COVID demonstrated death of GnRH neurons in all patients, thus suggesting that the GnRH neuronal network may play a role in the cognitive defects experienced by long COVID sufferers.^[^ ^168^ ^]^

The plasticity of the GnRH neuroendocrine system at the time of puberty makes it particularly vulnerable to external stressors. Although this may contribute to disordered puberty in patients with neurological conditions, it also offers a window of therapeutic intervention, whereby neuroendocrine circuits could be targeted to improve clinical outcomes.^[^ ^169^ ^]^ Endocrinopathies in patients with neurological conditions are often not prioritized within the acute clinical setting, but management has the potential to improve patient quality of life and reduce health burden in later life.^[^ ^29^, ^104^, ^105^ ^]^

Future directions for treatment of pubertal disorders in neurodevelopmental conditions may focus on the timing of therapeutic intervention, namely, during the period of minipuberty. Initiation of hormone therapy or other therapeutic interventions during minipuberty may lead to improved long‐term reproductive, behavioral, and cognitive impact, as suggested by the aforementioned animal studies administering NO to *Nos1*‐deficient mice.^[^ ^48^ ^]^ It is established that boys with severe forms of CHH, who demonstrate phenotypic features such as cryptorchidism in infancy, have diminished responses to GnRH or combined gonadotropin replacement in adolescence or adult life. This has brought minipuberty as a therapeutic window into focus in the field of reproduction.^[^ ^155^, ^170^ ^]^ However, given the nascent development of the identification of minipuberty as a key therapeutic window, little literature currently exists for discussion.^[^ ^171^ ^]^ Advances in this area will rely on long‐term follow‐up of patients treated in infancy and are complicated by the requirement for controlled trials so that the specific impacts of hormone therapy in minipuberty can be distinguished from the impact of concurrent supportive therapy throughout childhood and adolescence.

## CONCLUSIONS

Neurodevelopment of the reproductive systems and neural circuits controlling social behavior and cognitive abilities are closely linked. Here we have provided an overview of the molecular mechanisms of these shared pathways, both in health and in neurodevelopmental conditions, which present with disordered puberty and atypical social behavior. Children with neurodevelopmental disorders are found to be at increased risk of both premature pubertal changes and pubertal delay or failure when compared to the healthy population. The prevalence of neurodevelopmental comorbidities observed in patients with disorders of puberty suggests a dysregulation of biological processes within both neuroendocrine and social communication brain circuits.

One of the key considerations of pubertal timing in neurodevelopmental disorders is to ensure the psychological impact of puberty is managed in children with atypical neurological development. It is also essential to identify the shared molecular etiology of neurodevelopmental disorders and pubertal disorders to better understand the underlying biological processes and identify molecular therapeutic targets. This can enable clinicians to make accurate diagnoses, select therapeutic interventions, and implement suitable treatment pathways.

## AUTHOR CONTRIBUTIONS

Sasha R. Howard and Carmen Agustín Pavón conceived the review. Jordan E. Read, Alexandru Vasile‐Tudorache, and Angel Newsome contributed to the writing of the manuscript. Sasha R. Howard, Carmen Agustín Pavón, and María José Lorente revised the manuscript.

## COMPETING INTERESTS

The authors declare no conflicts of interest.

### PEER REVIEW

The peer review history for this article is available at: https://publons.com/publon/10.1111/nyas.15246.
